# Flail arm syndrome due to duplication mutations in the SMN1 gene: A case report

**DOI:** 10.1097/MD.0000000000033565

**Published:** 2023-04-21

**Authors:** Han Luo, Shanshan Li, Bo Liu

**Affiliations:** a Department of Neurology, Shenzhen Longhua District Central Hospital, Shenzhen, Guangdong, P. R. China.

**Keywords:** amyotrophic lateral sclerosis, flail arm syndrome, man-in-the-barrel syndrome, SMN1 gene

## Abstract

Flail arm syndrome (FAS) only involves the upper limbs early stage and manifests as proximal weakness and atrophy of both upper limbs and decreased tendon reflexes. As a benign variant type of amyotrophic lateral sclerosis, FAS progresses slowly, with no lower motor neuron signs in the lower limbs or bulbar muscles within 12 months after onset.

**Patient concerns:**

A 49-year-old male patient was admitted to the hospital with a 15-month history of proximal weakness and muscle atrophy in both upper limbs. His other symptoms and signs were not obvious.

**Diagnoses:**

Gene test results indicated that there were duplication mutations in the exon 7 to 8 region of the SMN1 gene.

**Lessons:**

The abnormal duplication of exons 7 and 8 of the SMN1 gene in this patient may increase the risk of FAS. Further studies are needed to identify the dominant genes and genetic factors causing males to be susceptible to FAS.

## 1. Introduction

Flail arm syndrome (FAS) is also known as Vulpian-Bernhardt syndrome, brachial amyotrophic diplegia, and neurogenic man-in-the-barrel syndrome, among others. ^[1,2]^ FAS only involves the upper limbs early stage and manifests as proximal weakness and atrophy of both upper limbs and decreased tendon reflexes. If the onset of FAS is not symmetrical, then tendon reflexes are not symmetrical. In addition, dysphagia and dyspnea may occur in the late stage. Most scholars believe that FAS progresses slowly, with no lower motor neuron signs in the lower limbs or bulbar muscles within 12 months after onset. The prognosis of FAS is better than that of upper limb onset amyotrophic lateral sclerosis (UL-ALS),^[3]^ and most cases of FAS are sporadic.^[4]^ This paper shares the data of a patient with duplication mutations in the exon 7 to 8 region of the SMN1 gene to provide new insights into the genetic susceptibility and diagnosis of FAS.

## 2. Case presentation

A 49-year-old male patient was admitted to the hospital with a 15-month history of proximal weakness and muscle atrophy in both upper limbs. In the past 15 months, the patient experienced progressive aggravation of weakness in the proximal upper limbs, difficulty lifting, and muscle atrophy in the upper limbs, which was more evident in the left upper limb. He had no walking instability, urinary or fecal incontinence, or limb paresthesias. He had a history of cervical spondylosis for 1 year and denied a history of chronic diseases such as cerebral infarction, hypertension, and type 2 diabetes mellitus. A physical examination indicated clear consciousness, clear speech, symmetrical bilateral frontal lines, normal eye movements in all directions, pupils equal in size (3.0 mm in diameter, bilaterally) and reactive to light, no nystagmus, symmetrical bilateral nasolabial folds, no deviation of the angle of the mouth, negative Gower sign, muscle fibrillation in both upper limbs, atrophy of the scapular girdle muscles, supraspinatus, and palmar muscles, grade 4 muscle strength of the proximal left upper limb, grade 3 muscle strength of the middle left upper limb, grade 4 + muscle strength of the distal left upper limb, grade 4 + muscle strength of the proximal right upper limb, grade 4 muscle strength of the middle right upper limb, grade 5 muscle strength of the distal right upper limb, and grade 5 muscle strength of both lower limbs. The tendon reflexes and muscle tone of both upper limbs were decreased, while the tendon reflexes and muscle tone of both lower limbs were normal. Rossolimo sign was positive (+) in the right upper limb and negative (−) in the left upper limb. Hoffmann sign, Babinski sign, and Chaddock sign were bilaterally negative (−). The pain, tactile and deep sensations in the bilateral limbs were symmetrical. The patient had no nuchal rigidity and was negative (−) for Kernig sign in both lower limbs.

**Figure 1.**
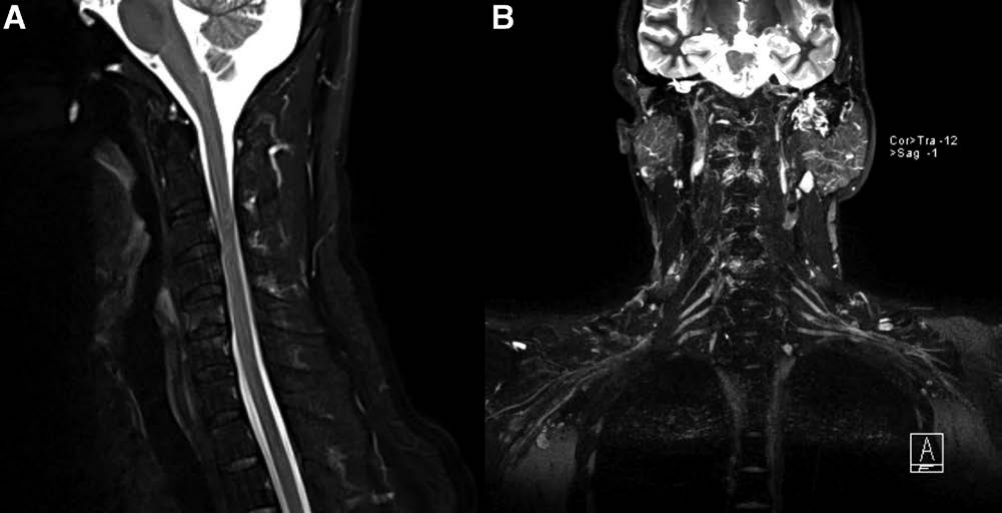
(A) Cervical spine MRI results: Cervical degenerative changes (C2/3–C6/7 intervertebral disc degeneration, mildly bulged C3/4–C6/7 intervertebral discs, C4–7 vertebral hyperostosis, and endplate inflammation between C5 and C6 vertebrae), (B) Bilateral brachial plexus MRI results: No evident bilateral brachial plexus injury.

**Figure 2.**
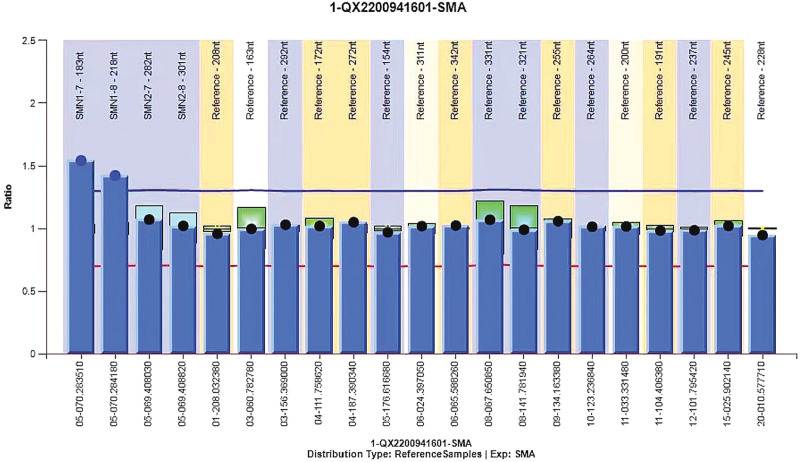
The copy numbers of exons 7 and 8 of the SMN1 gene and those of the SMN2 gene are 3, 3, 2, and 2, respectively.

Auxiliary examinations revealed the following: A muscle enzyme panel showed a creatine kinase concentration of 602↑ U/L and a creatine kinase isoenzyme concentration of 27.2↑ U/L. There were no obvious abnormalities in the anemia panel (vitamin B12, folic acid, and serum ferritin), the rheumatism arthritis panel (Rheumatoid factor, anti-RNP antibody, anti-SM antibody, anti-centromere antibody, anti-Jo-1 antibody, anti-SSA antibody, anti-SSB antibody, anti-Scl-70 antibody), the systemic lupus erythematosus panel (Antinuclear antibody, anti-double-stranded DNA antibody), the vasculitis panel (Anti-neutrophil cytoplasmic antibody, anti-neutrophil cytoplasmic antibody), and the tumor maker panel (alpha-fetoprotein, carcinoembryonic antigen, glycoprotein antigen 50, tumor specific growth factor). The brain, cervical spine, lumbar spine and brachial plexus MRI results indicated; Multiple ischemic foci of the bilateral frontal lobes; Hypertrophy of the bilateral inferior turbinates and slight inflammation of the bilateral ethmoid sinuses; Cervical degenerative changes (C2/3–C6/7 intervertebral disc degeneration, mildly bulged C3/4–C6/7 intervertebral discs, C4–7 vertebral hyperostosis, and endplate inflammation between C5 and C6 vertebrae) (Fig. 1A); Lumbar degenerative changes (mildly bulged L4/5 intervertebral discs and lumbar hyperostosis), and; No evident bilateral brachial plexus injury (Fig. 1B). Electromyography (EMG) revealed the following: the right biceps muscle showed a limited spontaneous potential during resting and increased amplitude and duration of motor unit potential (MUP) and decreased voluntary recruitment of motor units (MUs) during light contraction; the upper limbs showed no spontaneous potential during rest and increased amplitude and duration of MUP and decreased voluntary recruitment of MUs during light contraction; the lower limbs showed no spontaneous potential during rest and normal amplitude and duration of MUP and decreased voluntary recruitment of MUs during light contraction; the left sternocleidomastoid muscle showed no spontaneous potential during rest and increased amplitude and duration of MUP and normal voluntary recruitment of MUs during light contraction; and the right and left lumbar paraspinal muscles showed no abnormal changes. Gene test results indicated that there were duplication mutations in the exon 7 to 8 region of the SMN1 gene (The copy numbers of exons 7 and 8 of the SMN1 gene and those of the SMN2 gene are 3, 3, 2, and 2, respectively) (Fig. 2).

## 3. Discussion

### 3.1. Clinical features of FAS

The classic man-in-the-barrel syndrome (MIBS) refers to paralysis of both upper limbs caused by infarction of the anterior and middle cerebral arteries due to hypoperfusion, with normal motor function and no pathological reflex in the lower limbs, making patients appear as if their upper bodies are confined in a barrel. As such, Sage named this clinical syndrome MIBS in 1983.^[5]^ Motor neuron diseases manifesting as MIBS are more common in FAS and were first reported by Vulpian in 1886.^[1]^ According to relevant literature reports, the male-to-female ratio among FAS patients is 3:1 to 10:1.^[3,6,7]^ FAS onset occurs between the ages of 21 and 85 years (median age: 59.9 years; 95% CI: 50.3–68.1).^[6,7]^ Most scholars believe that FAS progresses slowly, with no lower motor neuron signs in the lower limbs or bulbar muscles within 12 months, and the prognosis is better than that of UL-ALS.^[8,9]^ In this paper, a case of a male patient with a disease course of 15 months is reported, and the only manifestations were atrophy of the upper limb muscles, scapular girdle muscles, and supraspinatus; there was no clinical or EMG evidence for damage to the lower limbs and bulbar muscles. Some scholars believe that not all patients have long survival^[10]^ and that differences may exist between different races. In addition, there is currently no gold standard for distinguishing between FAS, UL-ALS, and progressive muscular atrophy.

### 3.2. The etiology and diagnostic criteria of FAS

The etiology of MIBS includes amyotrophic lateral sclerosis (ALS), progressive muscular atrophy, facioscapulohumeral muscular dystrophy, cervical spinal cord infarction, and brachial plexus neuropathy, among others. The domestic and foreign literature has reported many cases of FAS-type ALS.^[11]^ FAS is a disease manifestation rather than the root cause of a disease, but such syndromes with characteristic manifestations are conducive to the exploration of their etiology. Patients with FAS have great individual differences in symptoms and disease progression; therefore, the diagnosis of FAS mainly depends on characteristic manifestations. Previous prospective studies, retrospective studies and reviews have summarized the characteristic manifestations of FAS for diagnosis as follows: Clinically significant signs of motor neuron damage (especially lower motor neuron damage) in the upper limbs that are consistent with a progressive development trend; Obvious atrophy and weakness of the proximal muscles of both upper limbs (except for cases with atrophy and weakness of the distal muscles of the upper limbs but without proximal involvement); Possible presence of signs of upper motor neuron damage (such as pathological reflexes or active deep reflexes of the upper limbs) but no increased muscle tone or myoclonus of the upper limbs during the disease course; and No clinically obvious lower motor neuron signs in other body parts within 12 months after the appearance of signs of motor neuron damage in the upper limbs (except for cases with evident lower motor neuron signs in the lower limbs or facial muscles before or at the same time as the appearance of lower motor neuron signs in the upper limbs).^[10,12,13]^

### 3.3. Differential diagnosis of FAS

The patient had a disease course of 15 months, and his clinical manifestations were difficulty lifting, muscle fibrillation in both upper limbs, and atrophy of the upper arm muscles, scapular girdle muscles, supraspinatus, infraspinatus, and palmar muscles. The EMG indicated neurogenic damage in both upper limbs, mainly involving proximal muscles, no involvement of the lower limbs, and no obvious bulbar damage, consistent with FAS. The main consideration was to distinguish FAS from progressive muscular atrophy, facioscapulohumeral muscular dystrophy, cervical spondylotic myelopathy, and brachial plexus neuropathy; Progressive muscular atrophy: Progressive muscular atrophy mainly involves the motor neurons of the anterior horn of the spinal cord. The disease onset mostly occurs between the ages of 40 and 50 years. The disease occurs more among males than among females and often progresses slowly. Its manifestations include muscle weakness, muscle atrophy, muscle fasciculation, and other signs and symptoms of lower motor neuron dysfunction in limbs. The first symptoms of progressive muscular atrophy are usually atrophy and weakness of the small muscles in 1 or both hands, gradually involving the forearms, upper arms, and scapular girdle muscles. Atrophy starting from the lower limbs is uncommon. Patients with progressive muscular atrophy typically have marked distal atrophy and decreased muscle tone and tendon reflexes but no sensory disturbance or sphincter involvement.^[14]^ This patient had evident proximal muscle atrophy, a positive Rosso limo sign in the right upper limb, and evidence of upper motor neuron damage, which are not fully consistent with the diagnosis of progressive muscular atrophy; Facioscapulohumeral muscular dystrophy: Facioscapulohumeral muscular dystrophy is a genetic muscle disorder that mainly affects the facial muscles and scapular girdle muscles and gradually affects the trunk and pelvic girdle muscles. Disease onset mostly occurs in adolescence, and disease progression is generally slow, without affecting the lifespan of patients. The first symptom in patients with facioscapulohumeral muscular dystrophy is shoulder muscle weakness, which manifests as a “winged scapula.” In the early stage of the disease, patients often show difficulty throwing. This patient had difficulty completing actions such as brushing his hair, washing his face, and lifting objects, primarily due to aggravation of proximal upper limb muscle weakness. He also had a slightly elevated serum creatine kinase level.^[15]^ However, the age and neurogenic damage (no myogenic damage indicated by EMG) of this patient did not support the diagnosis of facioscapulohumeral muscular dystrophy; Cervical spondylotic myelopathy: Cervical spondylotic myelopathy is mainly due to the degeneration of the intervertebral joints. Disease onset generally occurs between the ages of 40 and 60 years. Its manifestations may include motor or sensory dysfunction (such as radicular pain) and sphincter dysfunction. Although most patients with cervical spondylitis myelopathy have a long disease course, they may experience rapid disease progression. Strenuous exercise will aggravate the compression of the spinal cord and even affect the function of the spinal cord in severe cases.^[16]^ This patient had no obvious radicular pain or sensory disturbance. Hence, the clinical evidence did not support the diagnosis of cervical spondylotic myelopathy; Brachial plexus neuropathy: Most of the anterior branches of the C5 to T1 spinal nerves constitute the brachial plexus, which travels through the interscalene space and then the subclavian artery to enter the axillary cavity through the back of the clavicle. The common clinical manifestations of brachial plexus neuropathy are drooping upper limbs, adducted upper arms, inability to abduct or externally rotate the upper arms, adducted, and straightened forearms, inability to pronate, supinate or bend the forearms, preserved hand and finger motor functions, and constant sensory disturbances in the scapulae, upper arms, and lateral forearms. Similar symptoms to FAS include bilateral brachial plexopathy.^[17]^ However, brachial plexus neuropathy still could not explain the lack of sensory disturbance, and the progressive aggravation of symptoms in this patient. FAS can be easily distinguished from multifocal motor neuropathy, anterior spinal artery syndrome, Hirayama disease, paraneoplastic syndrome, and neuropathy caused by connective tissue disease. Because FAS patients aged 40 to 60 years are often complicated with cervical degenerative changes and spinal cord compression, they are easily misdiagnosed with cervical spondylotic myelopathy. If these patients receive surgery for cervical spondylotic myelopathy, they will suffer more pain and economic loss. In contrast, the misdiagnosis of other curable diseases or slow-progressing diseases as FAS can delay treatment. Therefore, it is necessary to strengthen our understanding of FAS, Furthermore, the relatively slow progression of the disease also helps patients eliminate the fear of ALS.

### 3.4. Gene expression heterogeneity in FAS

As a benign variant type of ALS, FAS is a common neurodegenerative disease that selectively damages the anterior horn cells of the spinal cord and motor neurons, resulting in progressive limb paralysis and bulbar palsy. Approximately 90% of ALS cases are sporadic, and familial aggregation of ALS is not evident; the interaction of genetic and environmental risk factors is considered the cause of increased susceptibility to the disease.^[18]^ However, some genetic tests have shown that h anti-RNP antibody A1 mutations may be associated with the etiology of FAS and that FAS with h anti-RNP antibody A1 mutations can sometimes show familial aggregation.^[19]^ Homozygous mutations in SMN1 cause spinal muscular atrophy (SMA) and are regulated by SMN2 copy number, but in a minority of cases, SMA is caused by point mutations in SMN1 rather than by homozygous deletion.^[4,20,21]^ A study found that duplication or deletion mutations in the SMN1 gene encoding the SMN protein among ALS patients lead to the skipping of exon 7 during the splicing of SMN2 pre-mRNA, resulting in a truncated mRNA that is translated into a very unstable protein (SMNΔ7), and that the loss of SMN protein function plays an important role in ALS, SMA, and other motor neuron diseases.^[22]^ Other studies have shown that abnormal SMN1 copy number is significantly associated with genetic susceptibility to ALS and that ALS patients with no SMN1 gene expression have longer survival,^[4,23]^ which is consistent with the relatively better prognosis of FAS. However, whether the abnormal duplication of exons 7 and 8 of the SMN1 gene in this patient increased the risk of FAS needs further clarification. At present, although there is no specific drug for the treatment of motor neuron diseases, the early diagnosis of rare diseases such as FAS can help determine the prognosis and reduce the waste of medical resources. Further studies are needed to identify the dominant genes and genetic factors causing males to be susceptible to FAS, and the development of genetic diagnosis and treatment technology will facilitate advances in etiology research, screening and treatment.

## 4. Conclusions

The abnormal duplication of exons 7 and 8 of the SMN1 gene in this patient may increase the risk of FAS. Further studies are needed to identify the dominant genes and genetic factors causing males to be susceptible to FAS.

## Author contributions

**Data curation:** Han Luo, Shanshan Li.

**Investigation:** Han Luo, Shanshan Li.

**Methodology:** Bo liu.

**Resources:** Han Luo.

**Visualization:** Bo liu.

**Writing – original draft:** Han Luo.

**Writing – review & editing:** Bo liu.
